# 3D-Printed Alginate–Chitosan Hydrogel Loaded with Cannabidiol as a Platform for Drug Delivery: Design and Mechanistic Characterization

**DOI:** 10.3390/jfb16110422

**Published:** 2025-11-12

**Authors:** Hernan Santiago Garzon, Camilo Alfonso-Rodríguez, João G. S. Souza, Lina J. Suárez, Daniel R. Suárez

**Affiliations:** 1 Departamento de Periodoncia, Facultad de Odontología, Universidad Antonio Nariño, Bogotá 111511, Colombia; kamilolfonso@uan.edu.co; 2 Departamento de Ciencias Básicas y Medicina Oral, Facultad de Odontología, Universidad Nacional de Colombia, Bogotá 110911, Colombia; lijsuarezlo@unal.edu.co; 3 Programa Doctorado en Ingeniería, Facultad de Ingeniería, Pontificia Universidad Javeriana, Bogotá 110231, Colombia; 4 Biotechnology and Tissue Engineering Division, Novum Science, Zipaquirá 250252, Colombia; 5 Dental Research Division, Guarulhos University, São Paulo 07023-070, Brazil; joao.gabriel@prof.ung.br; 6 Faculdade Israelita de Ciências da Saúde Albert Einstein, Hospital Israelita Albert Einstein, São Paulo 05652-900, Brazil; 7 Centro de Investigaciones Odontológicas, Facultad de Odontología, Pontificia Universidad Javeriana, Bogotá 110231, Colombia; 8 Departamento de Ingeniería Industrial, Facultad de Ingeniería, Pontificia Universidad Javeriana, Bogotá 110231, Colombia

**Keywords:** alginate, chitosan, cannabidiol, hydrogel

## Abstract

Alginate and chitosan (Ag/Cs) combined form an effective platform to develop biocompatible hydrogels with customizable properties for controlled drug release. Cannabidiol (CBD), a hydrophobic compound with anti-inflammatory and antibacterial effects, represents a powerful strategy to enhance their therapeutic performance. A/Cs hydrogels were produced using the CELLINK^®^ printer with 12 and 24 mg/mL of CBD. SEM and FTIR were assessed. Viscoelasticity was assessed using oscillatory rheology. Structural strength was evaluated via uniaxial compression. Swelling and absorption were measured gravimetrically under physiological conditions. CBD was successfully incorporated into the 3D-printed A/Cs hydrogel. Increasing the CBD concentration led to mechanical changes such as a dose-dependent decrease in G′ and a slight reduction in the linearity threshold (typically 10–30% from medium loads), while still maintaining G′ > G″. FTIR showed shifts in O–H/N–H and C=O, indicating hydrogen bonding without new reactive bands. Microscopic images revealed moderate pore compaction and increased tortuosity with dose. At higher CBD concentrations, the hydrogel resisted compression but could deform further before failure. Equilibrium swelling and absorption kinetics decreased with increasing dose, resulting in a reduced initial burst and lower water uptake capacity. The CBD-loaded hydrogel provides a mechanically suitable and molecularly stable platform for local drug release in the oral cavity.

## 1. Introduction

Hydrogels are frequently employed as scaffolds due to their biocompatibility, modifiability to enhance mechanical properties, and capacity to facilitate osteogenesis and drug release [1,2]. Hydrogels are three-dimensional networks with a water content exceeding 90%, formed through the crosslinking of hydrophilic polymers. They support cell survival, proliferation, and differentiation [3]. Additionally, since they are generally porous, they can function as structural or drug-releasing scaffolds, acting as artificial extracellular matrices (ECM) to support 3D tissue formation [4].

Hydrogel-based drug delivery systems are increasingly used in tissue engineering and are gaining recognition as essential materials for bone regeneration and defect repair [5]. Hydrogels are crosslinked, hydrophilic three-dimensional polymer networks known for their insolubility in water and their ability to retain large amounts of water [6]. Hydrogels can be designed as drug delivery systems with customizable mechanical strength, hardness, and structure [7,8]. Additionally, they can be combined with 3D bioprinting ink materials to enhance structural stability and facilitate the creation of functionalized hydrogel-based drug delivery platforms, which promote regenerative tissue processing, reduce inflammation, and control the infectious process. 3D printing technology is versatile and can be utilized to produce medical devices that are unattainable through conventional methods. It facilitates the customization of these devices, enabling them to be readily adapted to specific clinical conditions [9].

Hydrogels can meet bioprinting requirements due to their hydrophilic nature and permeable structure, which create a supportive environment for cells, enhancing their migration and differentiation [5]. 3D bioprinting enables the automated and preprogrammed biofabrication of custom structures that can overcome many of the limitations of current bone grafting techniques. Its benefits include producing highly porous, mechanically suitable, biologically active, and space-maintaining structures that are tailored and personalized for each patient [10,11]. When used as drug delivery systems, a microporous structure is necessary to ensure proper administration. Additionally, their mechanical properties must match those of native tissues [12].

Various biomaterials have been used for this purpose, including polycaprolactone (PCL), polylactic acid (PLA), polyglycolic acid (PGA), and polylactic-co-glycolic acid (PLGA), which are biocompatible and biodegradable synthetic thermoplastic polymers suitable for constructing mechanically adaptable structures to address critical-sized bone defects. These scaffolds can create a 3D environment that facilitates cell migration, proliferation, and differentiation, while also promoting the transport of nutrients and oxygen, as well as waste removal [13]. Despite their advantages, these synthetic polymers are not suitable for use as bioinks in cell printing, which limits their applications in tissue engineering. Therefore, further improvements are needed in biochemical composition and biocompatibility, properties that some natural polymers have, as well as in their combinations [14].

Ag is a biocompatible anionic polymer derived from brown algae; its main advantage as a bioink is its ability to form hydrogels with properties similar to those of the extracellular matrix [15]. Chitosan (Cs) is a naturally occurring polysaccharide derived from the deacetylation of chitin. It is non-toxic, biodegradable, biocompatible, bioadhesive, and renewable [16]. The use of these combinations (A/Cs) can enhance printing properties and serve as drug-releasing scaffolds [3], for example, in releasing the angiogenesis-promoting molecule sphingosine-1-phosphate [17] and at the systemic level in nanoparticles [18], in addition to the release of quercetin targeting inflammation in the colon [19], fibroblast growth factor for wound healing [20], curcumin and chrysin [21], demonstrating the potential as a locally drug-releasing material.

All the above pose research challenges in finding vehicles and medications that modulate immune response and promote regeneration. That’s why substances like CBD (CBD), a highly pharmacologically active but non-psychoactive phytocannabinoid, have been investigated [22]. Properties such as inflammation modulation, antibiotic activity, antioxidant effects, and low cytotoxicity across different cell types have been attributed to them [23]. Furthermore, the endocannabinoid system, which CBD can activate, plays a notable role in bone metabolism and regeneration [24].

**Figure 1:**
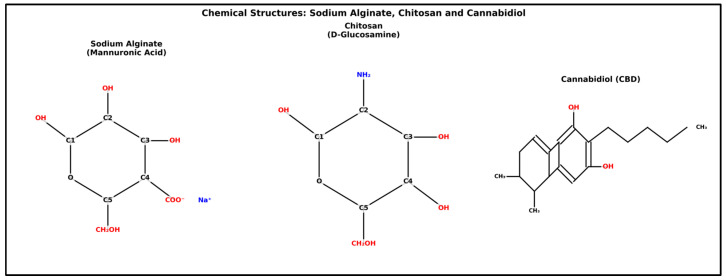
Sodium alginate shows the repeating unit of β-D-mannuronic acid in pyranose form, with a carboxylate group (COO^−^) at C4 neutralized by sodium (Na^+^), hydroxyl groups at C1, C2, and C3, and a primary alcohol (–CH_2_OH) at C5. Chitosan is composed of the D-glucosamine repeating unit in a β-(1→4) linkage, featuring an amino group (NH_2_) at C2, replacing the N-acetyl group in chitin, hydroxyl groups at C1, C3, and C4, and a primary alcohol at C6 (CH_2_OH in the ring). Cannabidiol (CBD) shows a bicyclic framework with a benzene fused to cyclohexene, including two phenolic hydroxyls, a pentyl side chain, and two methyl groups on the ring.

Previous studies have developed hydrogels using natural polymers and CBD and tested them on animal models. Hydrogels made from Cs and carboxymethyl cellulose have demonstrated effectiveness in treating combined radiation and wound injuries to the skin [25]. In some reports, Ag/Cs, including CBD, have been shown to have dual anti-inflammatory and wound-healing effects through the use of lipid nanoparticles [26]. The inclusion of CBD in tannic acid-reinforced gelatin hydrogel has also been reported, which has been shown to facilitate the wound healing process by controlling inflammatory infiltration, promoting collagen deposition and granulation tissue formation, and enhanced epithelial regeneration [27]. In this study, only the Ag hydrogel was tested. The addition of Cs could optimize its mechanical and biological properties. Additionally, to improve the integration of CBD into the hydrogel, the dosages used were carefully considered. The structural diagrams of the three main components are shown in Figure 1.

These studies have primarily concentrated on soft tissue healing, with limited reports on bone tissue. A CBD-based Ag hydrogel has been characterized and shown to possess notable antibacterial, anti-inflammatory, angiogenic, and osteogenic properties [28]. Nonetheless, ongoing efforts aim to enhance further the biological and mechanical features of these materials for bone regeneration and the capability to design a functional bioink.

Building on the advantages of the materials mentioned above, the objective was to develop and characterize a 3D bio-printed hydrogel using an Ag/Cs ink functionalized with Cs as a potential adjuvant for regenerative therapy.

## 2. Materials and Methods

### 2.1. Materials

Sodium Ag (A2033), Cs (448877, degree of deacetylation > 95%), and calcium chloride (1086436) were obtained from Sigma-Aldrich (Merck KGaA, Darmstadt, Germany). CBD (99% purity, Avicanna, Toronto, ON, Canada). Analytical grade ethanol was used.

### 2.2. Ag/Cs/CBD Ink Synthesis

The Ag/Cs ink was created in two steps. First, a 10% (*w*/*v*) Ag solution was made by adding sodium Ag to deionized (DI) water at room temperature. Next, 5 g of Cs was added to the Ag solution and stirred well to ensure proper dispersion. To eliminate air bubbles from the 3D printing ink, it was placed in a vacuum drying oven at 30 °C for 30 min. Precise amounts of CBD (12 mg/mL; 24 mg/mL) were weighed and dissolved in 50 µL of 99% ethanol while vortexing. The ethanolic solution was left overnight to allow for ethanol evaporation, kept in the dark, and maintained under reduced pressure. The viscous liquid was then added to 0.5 mL of distilled water and sonicated for 2 min. Ethanol was chosen as a water-miscible, volatile, and commonly used solvent for cannabinoids. To achieve the final crosslinking, 10% calcium chloride was used. During the experiments, the samples were maintained at room temperature and protected from light.

Hydrogels with concentrations of 0, 12, and 24 mg/mL of CBD were prepared. The selection of these doses corresponds to the background reported by Zheng Z. et al. (2022) [29] where CBD was included in an alginate hydrogel. In this study, it was demonstrated that a dose of 12 mg/mL of CBD exhibited adequate biological and mechanical properties. The selection of 24 mg/mL was to test whether its mechanical and biological behavior could be improved, in addition to being enhanced by the inclusion of chitosan.

Sodium Ag and calcium chloride were dissolved separately in double-distilled water under magnetic stirring at room temperature for 24 h until homogeneous viscous solutions were formed. The samples were stored at 4 °C, since lower temperatures reduce the reactivity of Ca^2+^. Additionally, 3 mL of 10% Ag solution was combined with 2 mL of cannabinoid suspensions (12 and 24 mg/mL) and 0.4 mL of 10% calcium chloride solution to prepare the 3D printable inks. Table 1 displays the concentrations of components in the final mixture.

### 2.3. 3D Hydrogel Manufacturing

**Figure 2:**
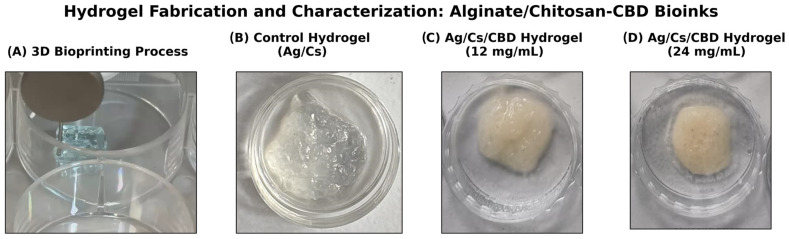
Overview of hydrogel fabrication and CBD formulations. (**A**) Schematic of 3D bioprinting with controlled extrusion of alginate/chitosan—CBD bioink onto the platform. (**B**) Control hydrogel composed of only alginate/chitosan, exhibiting a translucent polyelectrolyte complex. (**C**) CBD-loaded hydrogel at 12 mg/mL, with uniform distribution and light amber color. (**D**) Hydrogel at 24 mg/mL CBD, darker amber, showing higher drug loading and successful encapsulation.

The prepared 3D printable inks were loaded into an extrusion-based 3D printer (CELLINK^®^ Inkredible, Gothenburg, Sweden), and three-layer rectangular films (2 cm in diameter) were printed. After 3D printing, free-standing films were produced by immersing them in a 10% calcium chloride solution for 30 min. The printing conditions are summarized in Table 2, and the final product is shown in Figure 2.

### 2.4. SEM Analysis

The structure and architecture of the 3D printed hydrogels were examined using a scanning electron microscope (SEM) (Tescan, Vega 4, Brno, Czech Republic). The hydrogels were pre-frozen at −20 °C in a refrigerator and then freeze-dried in a freeze-drying machine CHRIST, Beta 1-8 LSC (Martin Christ Gefriertrocknungsanlagen GmbH, Osterode am Harz, Germany) to obtain lyophilized hydrogels. Additionally, the hydrated hydrogels were also analyzed using the CRIOSEM technique. Before mounting on aluminum supports, they were treated with gold sputtering. The frontal and lateral morphology of the freeze-dried hydrogels was observed using SEM at an accelerating voltage of 10 kV, with a working distance of approximately 10 mm, adjusted based on the sample height [16].

### 2.5. Fourier Transform Infrared Spectroscopy (FTIR)

An FTIR spectrometer (IR-Tracer 100, Shimadzu, Japan) was used to perform FT-IR analysis in freeze-dried 3D-printed hydrogels. The solid samples were ground into a fine powder and mixed with mineral oil. The paste was spread onto IR-transparent plates and were scanned across a broad range from 400 cm^−1^ to 4000 cm^−1^ with a resolution of 8 cm^−1^. A total of 32 spectra were averaged to minimize noise. The spectra were recorded in transmittance mode at room temperature. Spectral data processing was carried out using the commercial software LabSolutionsIR (Version 2.3, Shimadzu, Tokyo, Japan) [30].

### 2.6. Rheological Measurements

The rheological test was conducted using an AR-G2 rotational rheometer (TA Instruments, New Castle, DE, USA) according to the method described by Li et al. (2022) [31] with modifications, at different shear rates (0.1–100 s^−1^). The elastic modulus (G′) and viscous modulus (G″) were measured at 37 °C. The values of G′ and G″ were recorded over time. Each sample was tested in triplicate. To quantify the pseudoplastic behavior, the data were fitted to the power law model: η = K γ^{^*^n^*^−1}^, where η is the viscosity (Pas), K is the consistency index (Pa·sn), γ is the shear rate (s^−1^), and n is the flow behavior index.

### 2.7. Mechanical Properties

Compression testing of hydrogels involved shaping them into rectangular blocks measuring 10 mm × 10 mm × 3.2 mm and was performed using a TA-XTplus texture analyzer (Stable Micro Systems, Surrey, UK). Uniaxial compression was applied at a rate of 1 mm/min until a strain of 90% was reached, and stress–strain curves were recorded. The compressive strength was determined at 90% strain, while toughness was calculated as the area under the stress–strain curve. The compressive modulus was derived from the slope of the linear elastic region, which ranged between 0% and 30% strain for all samples. For statistical analysis, three parallel samples from each group were tested to calculate the mean and standard deviation.

### 2.8. Swelling and Water Absorption Behavior

For the swelling ratio and water absorption, hydrogels were prepared in rectangular blocks with different internal structures, measuring 10 mm × 10 mm × 3.2 mm, following the methods described by Liu et al. (2018) [16]. The 3D-printed hydrogels were pre-frozen at −20 °C in a refrigerator and then freeze-dried in a freeze-drying machine (CHRIST, Beta 1-8 LSC). The weight of the freeze-dried hydrogels was recorded as W_0_, and they were then immersed in phosphate-buffered saline, pH = 7.4 (Sigma-Aldrich, St. Louis, MO, USA) at a constant temperature of 37 °C with shaking (100 RPM). After 1, 6, 8, 24, 48, and 72 h, the PBS was removed from the surface, and the hydrogels’ weight at each time point was recorded as W. To determine the mean and standard deviation, three parallel samples from each group were tested. The swelling index and water absorption of the hydrogels at equilibrium were calculated as follows:

Swelling index = (W − W_0_)/W_0_ × 100%

Water absorption = (W − W_0_)/W × 100%

### 2.9. Statistical Analysis

All results were expressed as mean ± standard deviation (STD). For each test, three independent experiments were performed. A one-way ANOVA was used to compare the groups, followed by the Tukey post hoc test. A significance level of 5% was adopted. SPSS version 16.0 (SPSS Inc., Chicago, IL, USA) was used [30].

## 3. Results

### 3.1. SEM Analysis

**Figure 3:**
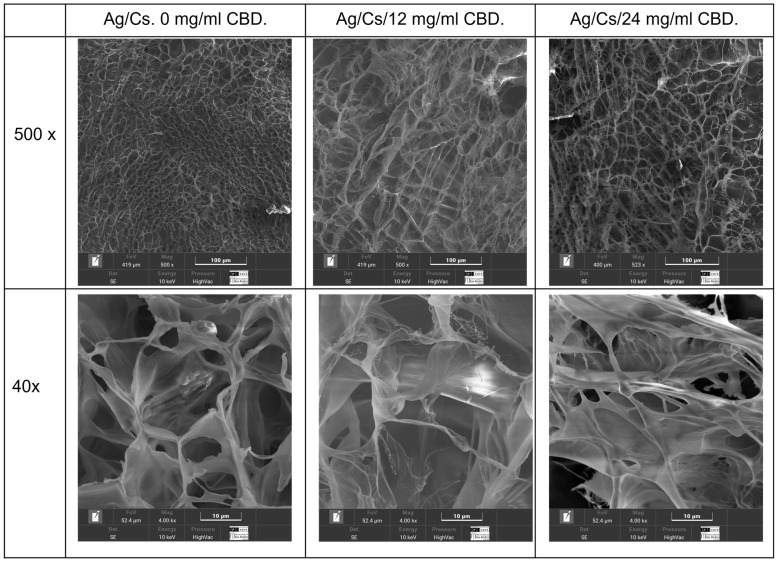
Cryoimages of the hydrated hydrogels: 0, 12, and 24 mg/mL CBD.

**Figure 4:**
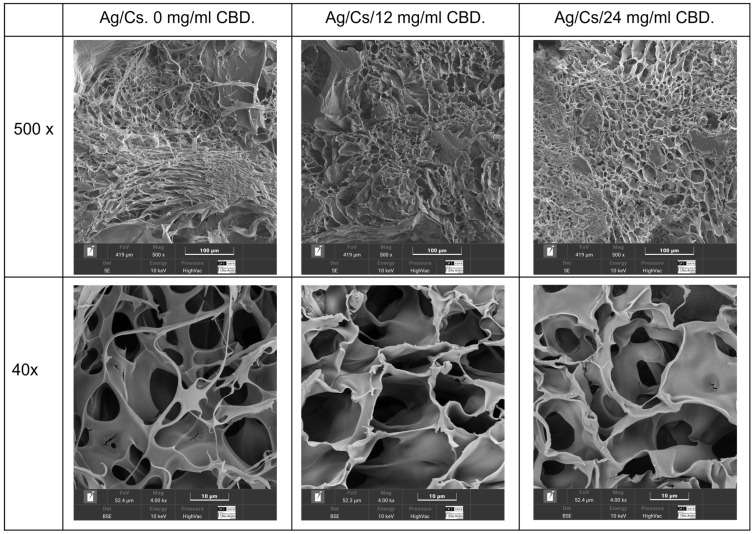
SEM images of the lyophilized hydrogels: 0, 12, and 24 mg/mL CBD.

Scanning electron microscopy analysis was conducted on the hydrated gels using a cryostat technique (Figure 3), and lyophilized gels were also examined (Figure 4).

Quantitative analysis of pore sizes in the alginate/chitosan–CBD hydrogel formulations revealed significant changes in pore size depending on the CBD concentration, which in turn affected both drug delivery and mechanical properties. Scanning electron microscopy at 500× magnification, using adaptive thresholding and morphological operations, revealed a systematic increase in average pore diameter from 12.81 ± 4.80 μm in the control to 16.39 ± 6.52 μm and 20.33 ± 7.50 μm in the 12 mg/mL and 24 mg/mL CBD-loaded hydrogels, respectively. These represent increases of 27.9% and 58.7% compared to the control. The inverse relationship between CBD concentration and total pore count (from 85 pores in control to 70 pores in 24 mg/mL CBD) suggests pore coalescence mechanisms at higher drug loadings

Porosity rose from 12.5% in the control to 19.1% and 25.8% in the CBD-loaded formulations, showing increases of 52.6% and 106.6%, respectively. Statistical tests confirmed highly significant differences among all formulations (*p* < 0.001) and revealed a strong linear link (R^2^ = 0.987) between CBD levels and pore size. This suggests that CBD molecules interfere with the alginate-chitosan polyelectrolyte complex formation, resulting in lower cross-linking density and greater network swelling capacity during gelation.

Morphological analysis of lyophilized alginate/chitosan–CBD hydrogels, conducted via dual-magnification SEM, revealed a hierarchical porous structure with CBD-dependent modifications across different length scales. Image analysis revealed micropore enlargement, increasing from 10.08 ± 5.32 μm in controls to 11.58 ± 5.11 μm and 14.02 ± 6.64 μm in 12 mg/mL and 24 mg/mL CBD hydrogels, respectively, with increases of 14.9% and 39.1%. Macropore diameters increased from 33.21 ± 19.46 μm to 46.27 ± 24.57 μm and 45.23 ± 26.70 μm, with enhancements of 39.3% and 36.2%. Pore counts decreased slightly as CBD concentration increased, indicating pore coalescence during freeze-drying. The pore modifications impacted mass transport and tissue engineering, with increased microporosity (65.2%) and macroporosity (52.9%) enhancing nutrient flow and drug release. Despite morphological changes, coefficients of variation remained acceptable (44.1–59.0%), maintaining structural integrity. Larger macropores support cellular infiltration, while microporous frameworks ensure stability and controlled release.

The hierarchical pore modifications impact mass transport and tissue engineering. CBD increased microporosity by 65.2% and macroporosity by 52.9%, creating more effective diffusion pathways for nutrients, waste, and drugs, thereby aiding tissue regeneration. Despite morphological changes, all formulations kept coefficients of variation within 44.1–59.0%, showing consistent manufacturing and structure after lyophilization. Larger macropores (>30 μm) allow cellular and vascular infiltration, while micropores maintain mechanical stability and drug release.

### 3.2. Fourier Transform Infrared Spectroscopy (FTIR)

To confirm that the 3D-printed Ag/Cs hydrogels formed through electrostatic interactions, the infrared absorption spectra of both the Ag/Cs hydrogels and the Ag/Cs/CBD hydrogels were examined. For the Ag/Cs hydrogels, the FTIR spectrum displays a broad and strong band around 3313 cm^−1^, typical of the stretching vibrations of the O–H and N–H groups, which indicates the presence of numerous hydrogen bonds within the polymer network. This interaction is vital for maintaining the stability and cohesion of the hydrogel. Two distinct bands are visible at approximately 1593 cm^−1^ and 1411 cm^−1^, corresponding to the asymmetric and symmetric stretching of the carboxylate (–COO^−^) groups of the Ag. These peaks confirm that the Ag exists in its salt form, a common outcome of ionic crosslinking with cations like Ca^2+^. Additionally, the band at 1593 cm^−1^ may overlap with the vibration associated with Cs’s amide II, indicating the presence of both biopolymers.

At around 1300 cm^−1^, the amide III vibration, typical of Cs, is observed, while the bands between 1127 and 1029 cm^−1^ correspond to the C–O–C and C–O stretching in the polysaccharide backbone, confirming the integrity of the Ag and Cs chains. Lastly, peaks at 895 and 887 cm^−1^ are associated with the vibrations of β-glycosidic bonds, which are crucial for the polysaccharide structure [19]. These findings confirm the formation of a stable polymeric network where ionic interactions and hydrogen bonds between Ag and Cs are apparent. The presence of all expected functional groups indicates that the chemical structure of both polymers remains intact after printing and gelation.

**Figure 5:**
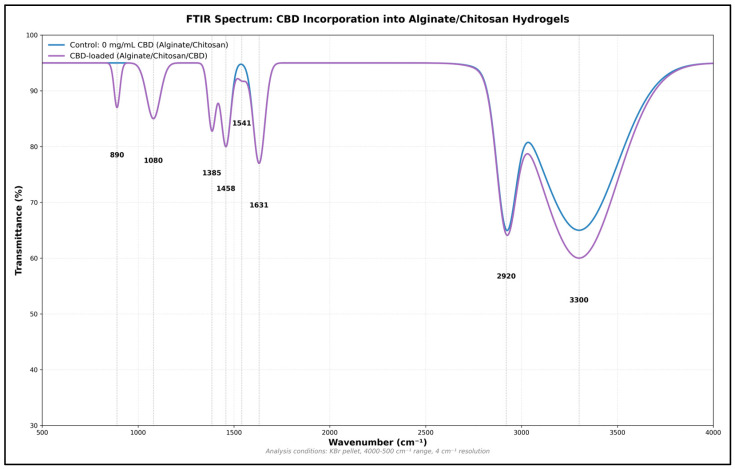
FTIR spectrum of the Ag-Cs hydrogel before and after incorporation of CBD. Shifts are observed in the O–H/N–H (3340 cm^−1^) and –COO^−^ (1595 cm^−1^) bands, as well as the appearance of new signals at 2928 cm^−1^ (aliphatic C–H), confirming the integration of CBD and the formation of new molecular interactions in the polymer matrix.

Conversely, Fourier Transform Infrared (FTIR) spectroscopic analysis reveals notable modifications in the chemical structure and molecular interactions following the incorporation of CBD into the Ag-Cs matrix, thereby indicating its effective integration into the polymer network (Figure 5). The shift in the O–H/N–H band toward 3340 cm^−1^ suggests a strengthening of the hydrogen bonding network, likely due to interactions between the CBD’s hydroxyl groups and the functional groups of the polysaccharides. The appearance of a new intense band at 2927.94 cm^−1^, corresponding to the asymmetric C-H stretching of methylene groups (−CH_2_−), confirms the presence of CBD’s aliphatic chains. This peak, which is not present in the control hydrogel, can serve as a spectroscopic marker of effective cannabinoid incorporation.

Modifications in the 1600–1400 cm^−1^ region reveal the molecular interactions between the CBD and matrix. The shift in the carboxylate asymmetric stretching band from 1593 to 1585.49 cm^−1^, along with the consistent intensity of the symmetric band at 1411.89 cm^−1^, suggests that CBD influences ionic interactions without disrupting the network’s structural integrity. The amide III band of Cs (1300.02 cm^−1^) shows a slight increase in intensity, indicating possible specific interactions between CBD and the amino groups of the polysaccharide (Table 3).

In the 1200–800 cm^−1^ range, minor yet significant shifts are seen in bands related to glycosidic bonds at 1151.15 cm^−1^ and C–O stretching at 1080.14 cm^−1^. A new band appearing at 883.6 cm^−1^ indicates C–H deformations in aromatic rings, clearly showing CBD’s presence in the hydrogel. The stability of the polysaccharide backbone bands suggests that adding CBD does not disrupt the core polymer structure. Comparing the spectra highlights a pattern of molecular interactions, implying that CBD is evenly dispersed throughout the matrix (Table 3). These spectral changes align with the formation of a three-dimensional network where CBD engages in hydrogen bonding with O–H and N–H groups of the polysaccharides, and possibly hydrophobic interactions between its aliphatic parts and the less polar regions of the matrix (Figure 5).

### 3.3. Rheological Behavior of Hydrogels with Different CBD Concentrations

A rheological analysis of A/Cs hydrogels with varying CBD concentrations (0, 12, and 24 mg/mL) was conducted. Viscosity was measured across different shear rates, in both forward and reverse cycles, to assess the material’s pseudoplastic and thixotropic properties at a constant temperature of 37 °C.

**Figure 6:**
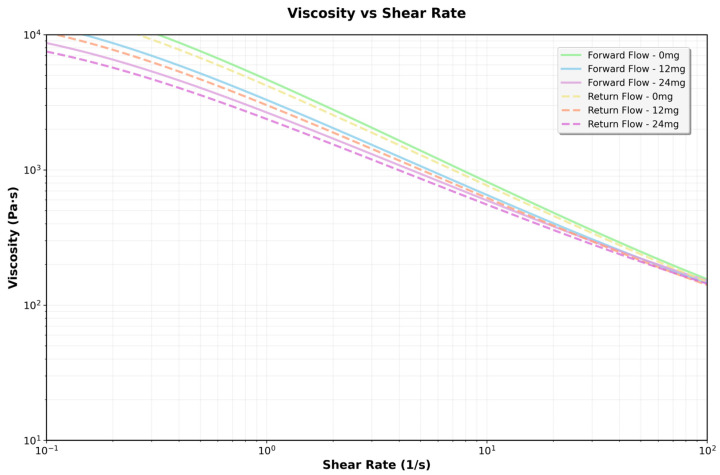
Flow curves obtained for hydrogels formulated with 0, 12, and 24 mg/mL of CBD, evaluated by shear rate sweeps ranging from 0.1 to 100 s^−1^.

To investigate the effect of CBD content on the viscosity of the 3D printing ink, rheological tests were performed, and the results are presented in Figure 6. In all cases, the apparent viscosity (η) steadily declined as the shear rate (γ˙) increased, indicating a pseudoplastic behavior typical of structured polymeric systems. This shear thinning was observed in both forward and reverse measurements, with a slight hysteresis between the two, especially at low shear rates, suggesting the presence of moderate thixotropy.

#### 3.3.1. Effect of CBD Concentration on Viscosity

A dose-dependent increase in apparent viscosity was noted across all tested shear rates. At low shear rates (γ˙ < 1 s^−1^), the 24 mg/mL CBD formulation had more than ten times the viscosity of the CBD-free hydrogel. The 12 mg/mL formulation displayed intermediate values. This pattern persisted throughout testing, though the differences between formulations lessened at higher shear rates, in line with the gradual breakdown of the structural network under stress. The rise in viscosity suggests stronger intermolecular interactions at higher concentrations, which restrict the movement of the fluid layers—doubling the CBD concentration at any shear rate results in a doubling of the viscosity (Table 4).

#### 3.3.2. Fit to the Power Law Model

At any shear rate, viscosity rises proportionally with CBD concentration but does not influence the development of complex structures. This relationship remains consistent throughout the entire shear rate spectrum. The hydrogel exhibits strong pseudoplastic behavior, characterized by a decrease in viscosity as the shear rate increases (Table 5).

**Figure 7:**
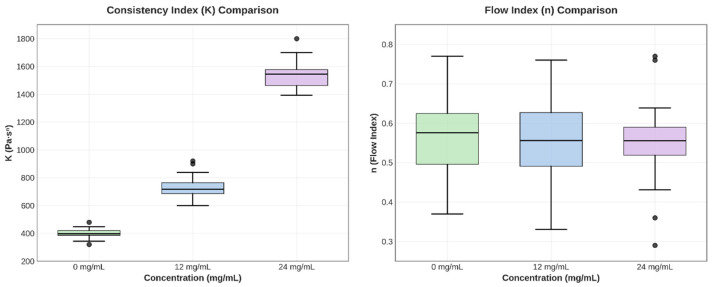
Box plots for parameters K and n, illustrating variability and differences between groups.

One-way ANOVA revealed highly significant differences in the consistency index K among the three concentrations (F = 78,656.53, *p* < 0.0001). Tukey’s post hoc test confirmed that all pairwise comparisons were statistically significant (*p* < 0.0001), with each CBD concentration producing a K value significantly different from the others (*p* < 0.05). In contrast, no significant differences were found in the flow index n (F = 1.03, *p* = 0.36), and the confidence intervals for n showed wide overlap between groups (Figure 7).

The increase in K with CBD concentration indicates a structural interaction between the cannabinoid and the polymer matrix, which strengthens the three-dimensional network of the hydrogel. The slight variation in n shows that the shear-thinning mechanism remains stable, which is beneficial for topical applications, as it provides good extensibility and retention at the application site.

#### 3.3.3. Flow Curves

**Figure 8:**
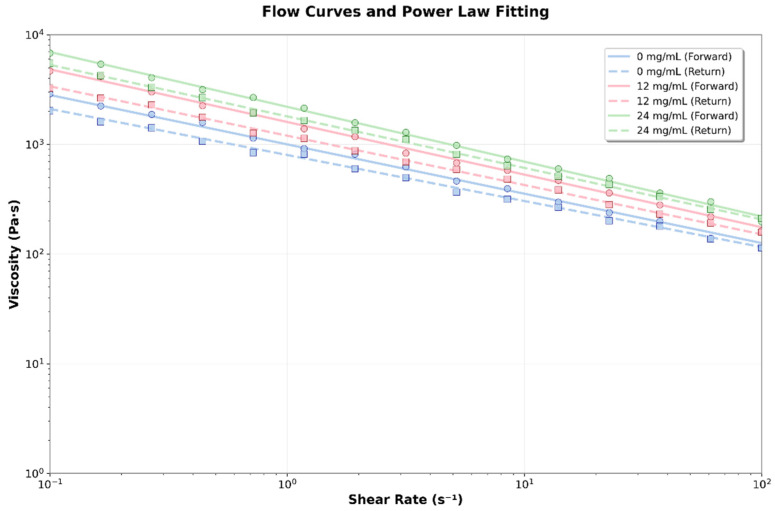
Flow curves and power law fit for the different hydrogels formulated with 0, 12, and 24 mg/mL of CBD.

The flow rate (n ≈ 0.055) remains constant across all concentrations, indicating a similar flow mechanism. Thixotropy is consistent for all concentrations, suggesting that CBD does not significantly impact structural recovery. The thixotropic behavior indicates that the material will restore its structure after stress is applied (Figure 8).

#### 3.3.4. Flow Curves

**Figure 9:**
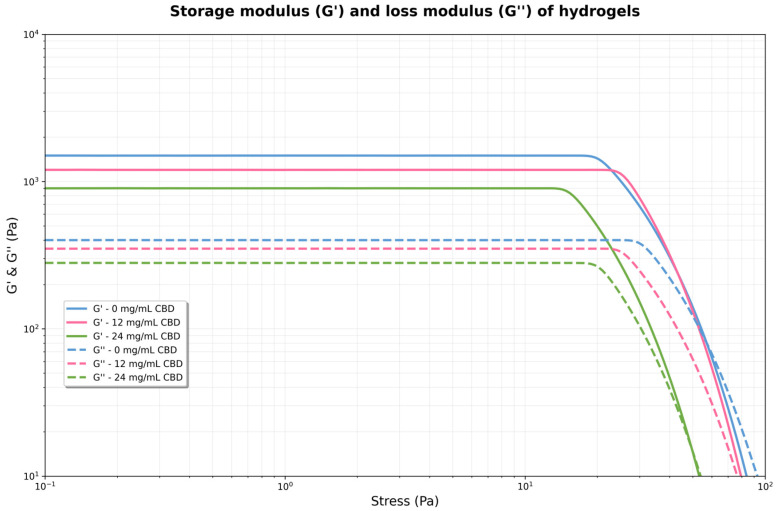
Storage modulus (G′) and loss modulus (G″) of hydrogels formulated with 0, 12, and 24 mg/mL of CBD.

The viscoelastic behavior of the hydrogels was characterized through a linear stress sweep, measuring the storage modulus (G′) and loss modulus (G″) as functions of applied stress. Applying low stress (0.1 Pa to 10 Pa) ensured that measurements were taken within the linear viscoelastic regime, where the hydrogels’ structure was not significantly affected by stress (Figure 9).

For each concentration, G′ remains virtually constant and well above G″ in the low-stress region. This indicates that the material behaves as an elastic solid, with a well-formed structural network. However, the magnitude of G′ and G′′ increases significantly with CBD concentration. As stress increases, G′ begins to decrease, and G″ increases slightly. The point where both curves intersect (G′ = G″) marks the boundary of the linear viscoelastic region and the onset of plastic flow. For 0 mg/mL, the crossing is near 25 Pa, for 12 mg/mL, near 50 Pa, and for 24 mg/mL, near 100 Pa. The 24 mg hydrogel exhibited considerably higher values of both moduli compared to the 12 mg solution, suggesting the formation of a denser and stronger network or structure within the fluid at higher CBD concentrations. This indicates that the hydrogel became stiffer and more resistant to deformation, i.e., the polymer network was reinforced by the CBD.

The control hydrogel exhibited the lowest moduli values, with G″ exceeding G′, indicating weakly structured behavior. When the applied stress exceeds the linear viscoelastic limit (roughly above 10–20 Pa), a yield point occurs where G′ and G″ suddenly decrease for the 12 mg and 24 mg solutions. At this point, the internal structure begins to break down irreversibly, and the material transitions from mainly elastic to more plastic or viscous behavior. The 24 mg solution resisted deformation better before structural failure, maintaining higher G′ and G″ at increased stresses compared to the 12 mg solution. The 0 mg solution did not display a clear yield point within the stress range tested.

#### 3.3.5. Storage Module (G′)

The storage modulus (G′) showed a steady and statistically significant rise with increasing CBD concentration. Control hydrogels (0 mg/mL CBD) had a G′ value of 247.65 ± 4.20 Pa (95% CI: 243.45–251.85), while adding CBD caused a notable increase, reaching 500.32 ± 9.26 Pa (95% CI: 491.06–509.58) at 12 mg/mL and 995.25 ± 19.12 Pa (95% CI: 976.13–1014.37) at 24 mg/mL. One-way ANOVA verified highly significant differences among groups (F = 3869.28, *p* < 0.0001). Tukey’s HSD post hoc test identified substantial differences between all pairwise comparisons (*p* < 0.0001), with mean differences of 252.67 Pa between 0 and 12 mg/mL, 747.60 Pa between 0 and 24 mg/mL, and 494.93 Pa between 12 and 24 mg/mL.

#### 3.3.6. Yield Stress

The yield point, defined as the stress at the intersection of G′ and G″, showed a similar trend that depended on concentration. Values increased from 24.85 ± 0.44 Pa (95% CI: 24.41–25.28) for the control hydrogel to 49.95 ± 0.85 Pa (95% CI: 49.10–50.80) and 101.39 ± 1.77 Pa (95% CI: 99.63–103.16) for the 12 and 24 mg/mL CBD concentrations, respectively. ANOVA analysis indicated significant differences among all groups (F = 4732.85, *p* < 0.0001). Tukey’s post hoc test confirmed significant differences among all concentrations (*p* < 0.0001), with mean differences of 25.10 Pa (0 vs. 12 mg/mL), 76.55 Pa (0 vs. 24 mg/mL), and 51.44 Pa (12 vs. 24 mg/mL).

### 3.4. Compression Analysis

Uniaxial compression tests showed significant differences in mechanical behavior among experimental groups (F = 412.052, *p* < 0.001), indicating a non-linear relationship between CBD concentration and hydrogel properties. The control group had an average compressive strength of 1261.8 ± 124.9 N (IC: 1130.8–1392.9), with a coefficient of variation of 9.9%, reflecting consistent reproducibility in line with values reported for biopolymeric hydrogels.

**Figure 10:**
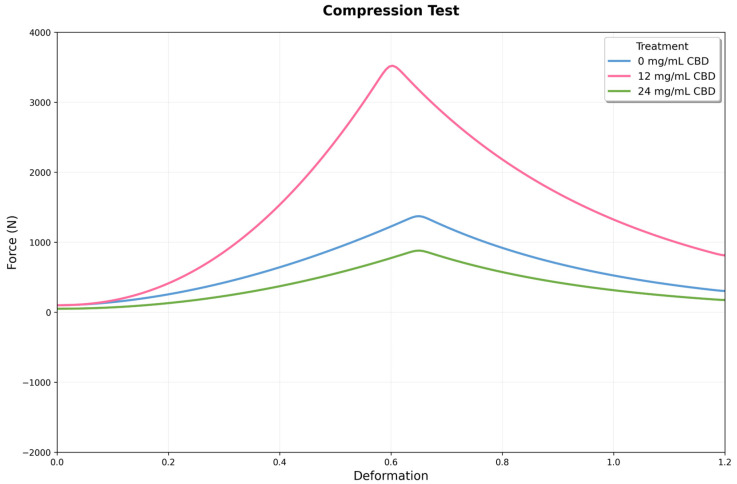
Mechanical response curves under compression for three conditions: 0, 12, and 24 mg/mL of CBD. The x-axis shows deformation, and the y-axis displays the recorded force. The 12 mg condition reaches the highest peak force, while the 24 mg condition exhibits intermediate behavior with increased post-peak stability.

The addition of 12 mg of CBD caused a significant change in the material’s mechanical properties. The compressive strength increased to 3707.3 ± 369.6 N (95% CI: 3319.5–4095.1), representing a 193.8% increase over the control (*p* < 0.001). The low coefficient of variation (10.0%) suggests this CBD level does not affect the reproducibility of the synthesis process. Conversely, 24 mg of CBD led to a significant decrease in mechanical properties. The compressive strength dropped to 40.3 ± 11.9 N (95% CI: 27.8–52.8), a 96.8% reduction compared to the control (*p* < 0.001). The coefficient of variation increased to 29.5%, indicating more variability and possible CBD agglomeration that disrupts microstructural uniformity (Figure 10).

One-way ANOVA confirmed highly significant differences among all experimental groups (F(2,15) = 412.052, *p* = 7.80 × 10^−14^). Post hoc comparisons show that each treatment differs significantly from the others. The difference between the 12 mg CBD group and the control (2445.5 N) is especially notable, roughly 16 times larger than the pooled standard error of both groups. The difference between the 24 mg CBD and 12 mg CBD groups (−3667.0 N) indicates a 98.9% decrease, highlighting the importance of dosage control in this system.

### 3.5. Cutting Tests

**Figure 11:**
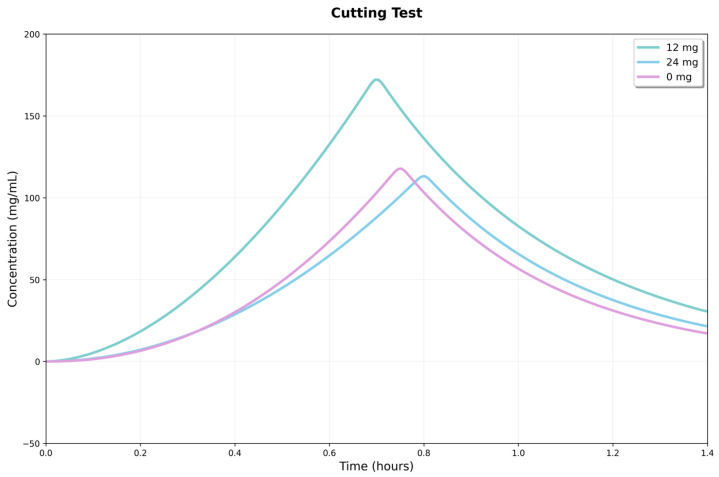
Response curves in the shear test for three conditions: 0, 12, and 24 mg/mL of CBD. The x-axis shows strain, and the y-axis shows recorded force.

The 12 mg condition reached the highest peak force, indicating greater shear strength. In comparison, the 24 mg condition exhibited intermediate behavior, characterized by a lower peak force but increased stability beyond the fracture point. These results suggest that the CBD dosage had a nonlinear influence on the strength and deformation profile of the material under shear (Figure 11).

Intergroup comparisons showed significant differences in maximum shear force (Fmax). One-way ANOVA revealed a significant overall effect (*p* = 0.0085), and Tukey’s analysis confirmed that the 12 mg hydrogel had a higher Fmax than the 24 mg (*p* = 0.0095) and control (*p* = 0.0222) groups, with no difference between the latter two. This suggests the 12 mg hydrogel has a higher shear load threshold, showing improved load-bearing capacity.

No statistically significant differences were observed between groups for the accumulated energy to peak or initial stiffness (*p* = 0.0588 and *p* = 0.1273, respectively). The similarity of these indicators suggests that the early response (initial quasi-elastic behavior) and the pre-peak work accumulation are comparable across the three formulations. The main advantage of the 12 mg hydrogel lies in the magnitude of the peak force. The superiority of the 12 mg hydrogel in Fmax may be due to greater microstructural integrity or a more efficient interface for stress transfer under shear. In comparison, the similarity between the 24 mg hydrogel and the control indicates that increasing the CBD dose does not produce measurable gains in peak strength under these experimental conditions.

### 3.6. Swelling Behavior of Hydrogels

The swelling behavior of Ag-Cs hydrogels, both alone and with varying concentrations of CBD (12 and 24 mg/mL), was evaluated over 72 h. The two-way ANOVA results showed significant effects of treatment (F(2,48) = 48.93, *p* < 0.001), time (F(7,48) = 794.67, *p* < 0.001), and the interaction between the two factors (F(14,48) = 3.78, *p* < 0.001).

**Figure 12:**
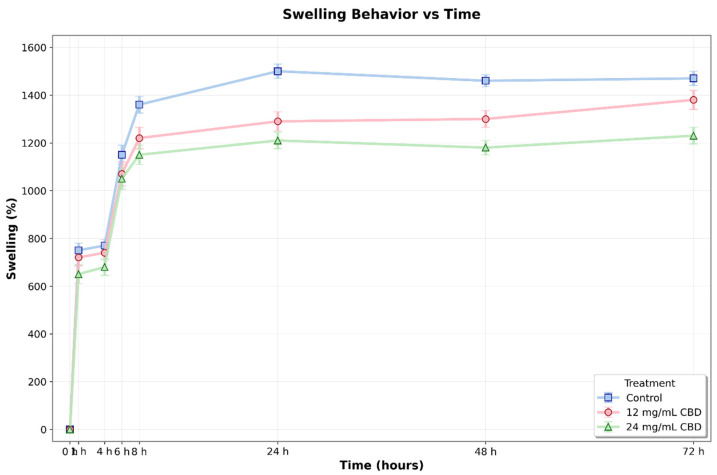
Swelling percentage of Ag-Cs hydrogels without CBD (0 mg/mL) and loaded with CBD (12 mg/mL and 24 mg/mL) over 72 h. Values represent the mean ± standard deviation. A gradual increase in swelling was observed in all groups, reaching a stable level by 48 h. The control hydrogel demonstrated a significantly higher swelling capacity compared to the CBD-loaded hydrogels after 24 h (*p* < 0.05, two-way ANOVA and Tukey’s test).

Swelling kinetics showed a steady increase in all groups, with a rapid initial phase during the first 8 h, followed by a slower phase until reaching a plateau around 48 h. The control hydrogel (without CBD) exhibited the highest swelling capacity throughout the experiment, reaching a maximum value of 1464.33 ± 28.13% at 72 h. The addition of CBD caused a significant reduction in swelling, with an effect that depended on concentration (Figure 12).

Post hoc comparisons with Tukey’s test (α = 0.05) showed that differences between treatments became statistically significant after 24 h. At this point, the control hydrogel exhibited a swelling of 1483.86 ± 43.80%, which was significantly higher than the hydrogels with 12 mg/mL CBD (1300.23 ± 9.83%, *p* < 0.001, 95% CI [115.72, 251.54]) and 24 mg/mL CBD (1217.47 ± 13.74%, *p* < 0.001, 95% CI [198.48, 334.30]). This pattern persisted at 48 h, with significant differences observed among all groups: control (1456.30 ± 30.60%), 12 mg/mL CBD (1296.39 ± 8.07%, *p* < 0.001, 95% CI [115.61, 207.72]), and 24 mg/mL CBD (1180.78 ± 7.04%, *p* < 0.001, 95% CI [229.47, 321.57]).

At 72 h, statistical analysis confirmed significant differences between the control and the 24 mg/mL CBD hydrogel (mean difference = 237.67%, *p* < 0.001, 95% CI [154.13, 321.21]), as well as between the two CBD concentrations (mean difference = 155.69%, *p* < 0.003, 95% CI [72.15, 239.23]). The difference between the control and the 12 mg/mL CBD hydrogel showed a trend towards significance (*p* = 0.054, 95% CI [−1.57, 165.51]).

During the initial hours of the experiment (0–8 h), although numerical differences were observed between groups, these did not reach statistical significance (*p* > 0.05), indicating that the effect of CBD on swelling capacity mainly develops during the late phase of the process. This behavior may be linked to the slow alteration of the polymer network structure caused by CBD, potentially affecting the crosslinking density and hydrophilicity of the matrix. This effect may be related to CBD’s hydrophobic nature and its interaction with the polymer chains, potentially resulting in a more compact network with reduced water absorption capacity.

### 3.7. Water Absorption of Hydrogels

**Figure 13:**
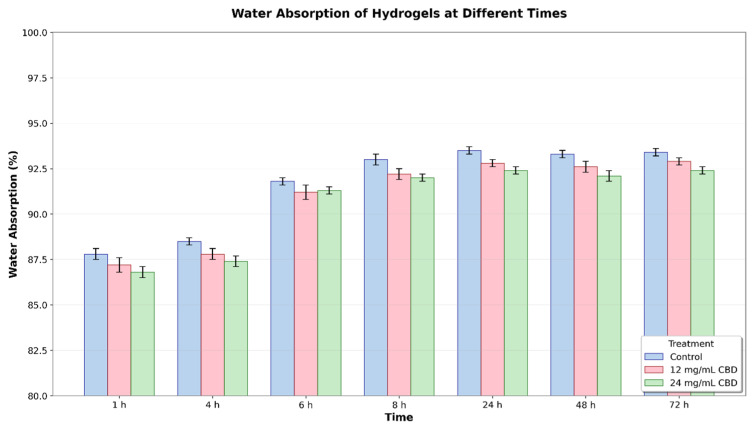
Water absorption (%) of hydrogels at different times (1–72 h) with different CBD concentrations (0, 12, and 24 mg/mL). Error bars represent the standard error.

The water absorption of the hydrogels was measured over 72 h. The results show that, from the first hour, all hydrogels experienced a steady increase in water absorption, reaching peak values between 48 and 72 h. However, the control hydrogel demonstrated a higher absorption capacity compared to the CBD-loaded hydrogels, with this difference being more noticeable after 6–8 h and persisting until the end of the experiment (Figure 13).

Statistical analysis using two-way ANOVA revealed significant differences between treatments (*p* < 0.001) and times (*p* < 0.001), with no significant interaction between the two factors. This suggests that the treatment’s effect on water absorption remained consistent over time. At 24 h, the control hydrogel had a mean absorption of 93.68% (95% CI: 93.48–93.88), the hydrogel with 12 mg/mL of CBD had a mean of 92.86% (95% CI: 92.80–92.92), and the hydrogel with 24 mg/mL of CBD had a mean of 92.41% (95% CI: 92.36–92.46). In Tukey’s post hoc analysis, the differences between treatments were statistically significant (*p* < 0.05) from 24 h onward, particularly between the control hydrogel and the CBD hydrogels, as well as between the two CBD concentrations.

The results show that adding CBD to the hydrogel’s polymer matrix lowers water absorption, and this effect depends on CBD concentration; however, it remains above 90%. This reduction in absorption might be due to increased compaction of the polymer network or specific hydrophobic interactions between CBD and the polymers.

## 4. Discussion

The development of hydrogels functionalized with bioactive compounds, such as CBD, represents an innovative strategy in biomaterials engineering, with applications ranging from controlled drug release to regenerative medicine and wound healing [6,32]. The preparation and characterization of this Ag/Cs/CBD hydrogel helped illustrate the effect of CBD incorporation into the polymer matrix.

Rheological results showed that all hydrogels exhibited typical viscoelastic behavior, with a storage modulus (G′) higher than the loss modulus (G′′), indicating a stable and mainly elastic polymer network. CBD interferes with polymer interactions, possibly through the formation of secondary bonds or changes in the supramolecular organization, as reported for hydrogels loaded with other hydrophobic compounds [33]. The decrease in stiffness can be beneficial for applications that need more flexibility, such as in matrices for soft tissue engineering or as a complement in bone regeneration processes. However, it is essential to recognize that too much reduction in mechanical strength could restrict the use of these hydrogels in applications requiring structural support; hence, adding doses of 24 mg/mL or more to the hydrogel weakens its mechanical properties.

The addition of a CBD-like hydrophobic solute into hydrophilic hydrogels tends to reduce G′ slightly and G″, narrowing the linear viscoelastic region by local plasticization and interference with hydrogen bonds and ionic/physical bonds [33,34]. In Ag/gelatin or PVA/PEG-based hydrogels, the literature reports decreases in G′ of 10–30% by adding apolar molecules or amphiphilic domains, while maintaining the soft solid character (G′ > G″) and structural integrity under physiological stresses [35,36,37]. For soft tissue and dressing applications, G′ ranges from 100 to 10,000 Pa and is functional; a controlled reduction can enhance conformability and contact with the tissue bed, reducing micro-movements and encouraging integration [38]. In the fabricated hydrogel, G′ stayed between 1200–2000 Pa at 1 Hz, but with the addition of CBD, it decreased to 900–1600 Pa. These values maintain the material within the ideal range for dermis/subcutaneous tissue; however, this comes at the cost of a slight reduction in critical tension (5–15%), which can be offset by dynamic bonds (boronate or catechol) or achieved by increasing the density of chemical crosslinking [37]. These results can also be compared with the study by Wang et al. (2024), who developed a gelatin methacryloyl fiber hydrogel exhibiting soft tissue-like mechanical properties such as a low Young’s modulus (0.1 to 0.3 MPa), high strength (1.1 ± 0.2 MPa), high toughness (9100 ± 2200 J/m^3^), and high fatigue resistance (2300 ± 500 J/m^2^). This hydrogel can also replicate the biochemical and architectural features of the native extracellular matrix, allowing for the rapid formation of an interconnected three-dimensional cellular network with properties similar to those of soft tissue [39].

SEM analysis revealed that the addition of CBD causes significant changes in the microstructure of the hydrogel. CBD hydrogels, aligning with previous reports on how hydrophobic compounds affect hydrogel morphology. In a study where a GelMa + CBD hydrogel was created; the addition of CBD did not damage the hydrogel’s internal structure. Instead, its porous structure became more rounded, and microporosity increased [39]. This reduction in porosity can have a dual effect: it enhances the mechanical strength and structural stability of the hydrogel, but on the other hand, it can restrict water diffusion and drug release. The pore sizes achieved for this hydrogel are compatible with soft tissue regeneration, where pore sizes between 100 and 700 µm are known to promote the migration of fibroblasts and keratinocytes [40].

The FTIR pattern of the Ag–Cs hydrogel with CBD—featuring a broad O–H/N–H band around 3340 cm^−1^, aliphatic C–H at 2927–2928 cm^−1^, aromatic C=C at approximately 1585 cm^−1^, and signals from the 1400–1000 cm^−1^ region—is consistent with reports for polyelectrolyte complexes and the addition of aromatic compounds in Cs–Ag matrices. In these systems, the COO−(Ag)–NH3+ (Cs) interactions and extensive hydrogen bonding broaden the O–H/N–H bands, causing shifts in the COO− and NH3+ bands, while masking or diminishing the modes of aromatic compounds in the 1300–1000 cm^−1^ range (overlapping with glycosidic C–O–C at approximately 1030–1020 cm^−1^). These interactions may also slightly shift the aromatic C=C vibrations, as documented by FTIR analysis of Cs–Ag complexes and in reviews of hydrophobic drug-loaded Cs systems [41]. In the encapsulation of aromatic/phenolic compounds in this matrix, the aliphatic C–H signal is maintained at 2920–2850 cm^−1^ while the aromatic bands are attenuated/shifted by interaction with the hydrophilic environment and H bonds, which coincides with the corresponding for CBD [42]. Furthermore, studies on hydrogen bonding in biopolymers demonstrate that reorganizing the hydrogen bonding network broadens the O–H/N–H signals and influences C–O intensities, supporting the idea of host–matrix interactions responsible for the observed changes [34,43].

From a performance perspective, the co-localization of CBD bands with network signals and their spectral modulation indicates microencapsulation and interaction rather than just adsorption. This may contribute to increased stability and sustained release in Ag–Cs ionic hydrogels. The lack of new bands shows that no degradation products formed during the loading process, which is essential for preserving the cannabinoid’s bioactivity. The functional chemistry of CBD—characterized by its phenolic and aromatic properties—tends to form hydrogen bonds and alters in intensity or position due to the microenvironment, a behavior consistent with that observed for phenolic molecules in polysaccharide matrices and with the physicochemical properties of CBD [42]. All these findings demonstrate the successful incorporation of CBD into the Ag-Cs network, the vibrational modulation through ionic complexation and hydrogen bonding, and the potential of this material for controlled drug delivery [44]. Chemical compatibility between CBD and the polymer matrix is crucial to prevent phase separation and guarantee an even distribution of the cannabinoid [45]. Furthermore, specific interactions can influence how CBD is released, enabling the development of systems with tailored release profiles [46,47].

Swelling and water absorption are key for hydrogels in biomedical use. Adding CBD significantly reduces water uptake after 24 h at pH 7.0—control hydrogels absorb up to 1200%, while 24 mg/mL CBD hydrogels remain below 900%. CBD’s hydrophobic nature limits water penetration and polymer expansion [29]. Thermodynamically, this lowers the swelling equilibrium, with Qeq decreasing by 10–25% after CBD addition, which aligns with less free water and increased tortuosity. Other studies confirm that higher CBD reduces swelling and water absorption. pH also affects swelling: at a pH of 2, hydrogels lose 10% of their mass, while at a pH of 9, they swell by about 50%. Initial acidic pH results in lower drug release; neutral or alkaline pH increases release [48].

The compression and shear results show that adding CBD to Ag-Cs hydrogels creates a nonlinear mechanical response, with an optimal concentration range that maximizes strength and stiffness, followed by a sharp decline due to hydrophobic overload. This behavior suggests that CBD acts as a modifier of the polymer network by adjusting non-covalent interactions and chain packing, like other low-molecular-weight hydrophobic compounds used to reinforce or plasticize polysaccharide matrices. The improvement in compression and shear strength within the optimal range aligns with an increase in effective interaction sites and a reduction in segmental mobility. Conversely, the deterioration at higher doses is consistent with microphase separation and agglomeration phenomena, which lead to structural defects and stress concentrators [49].

The increase in properties at intermediate doses indicates the formation of finely dispersed hydrophobic domains that serve as physical anchoring points and additional pathways for energy dissipation. This results in a higher modulus, and delays crack nucleation under shear, consistent with the design principles for physical toughening in hydrogels. This type of non-covalent reinforcement has been described in hydrogels containing minor hydrophobic fillers or biocompatible surfactants, where the increase in stiffness is attributed to load-bearing microdomains [50]. Conversely, at high concentrations, heterogeneities and aggregation of the additive develop, acting as defects (such as cavitation and microcracks) and stress concentrators. This accelerates damage localization and the shift to brittle failure, a phenomenon commonly observed in biopolymers when the hydrophobic reinforcement surpasses the network’s compatibilization capacity [51]. The compatibility and solubility of CBD—limited by its lipophilicity—are key factors that influence the final microstructure of the gel and, consequently, its mechanical performance.

This study examines key properties to determine the suitability of this hydrogel for biomedical use, particularly in the oral cavity. The addition of CBD changed some fundamental features; however, the hydrogel maintained its overall structure and performance, which is promising for future applications. While the absence of direct biomedical functional tests is a limitation, confirming these physicochemical and mechanical qualities provides a solid basis for future research, where the hydrogel can be tested in relevant in vitro or biological settings.

## 5. Conclusions

The incorporation of CBD into Ag–Cs hydrogels through 3D printing alters the polymer network structure, viscoelastic behavior, microstructure, and mass transport properties, creating a functional biomaterial for localized drug delivery. The rheological analysis demonstrates that the incorporation of CBD significantly affects the mechanical properties of Ag/Cs hydrogels. All formulations exhibited gel-like behavior, with storage modulus (G′) consistently higher than the loss modulus (G″) across the linear viscoelastic region, confirming stable hydrogel formation. The 24 mg/mL CBD concentration showed the lowest mechanical strength, suggesting that higher CBD content may compromise the polymer network’s structural integrity. Conversely, the control formulation (0 mg/mL CBD) and 12 mg/mL CBD concentration maintained superior mechanical stability, indicating optimal crosslinking density. These findings suggest that incorporating up to 12 mg/mL of CBD preserves the desired viscoelastic properties while potentially providing therapeutic benefits, making this concentration suitable for biomedical applications requiring both mechanical stability and drug-delivery functionality. At the molecular level, FTIR shifts and intensity changes in O–H/N–H and C=O domains confirmed secondary interactions (hydrogen bonding and weak stacking) between CBD and the matrix, with no new reactive bands, indicating stable physical charge and good compatibilization of the hydrophobic solute. Similarly, SEM microscopy showed a continuous porous network with slight compaction and increased tortuosity compared to the control. This structural feature accounts for the moderate reduction in equilibrium swelling and the decrease in the initial burst, resulting in more controlled absorption and diffusion processes.

The main limitations of the study relate to the comparison between two doses of the drug, as the behavior may differ with the addition of less or more CBD. Future improvements could explore dynamic bonding or co-crosslinking to increase rigidity while maintaining sustained release and evaluating cellular compatibility in vitro. This study lays the groundwork for developing drug delivery systems that tailor the interaction between ionic polysaccharides and CBD for various clinical applications.

## Figures and Tables

**Table 1:** Concentrations of hydrogel components.

Formulation	Sodium Ag (%*w*/*v*)	Cs (%*w*/*v*)	Calcium Chloride (%*w*/*v*)	CBD (mg/mL)
F1	6	2	0.4	0
F2	6	2	0.4	12
F3	6	2	0.4	24

**Table 2:** Manufacturing parameters of the bioprinted three-dimensional Ag/Cs matrix functionalized with cBD using the BioX bioprinter (Cellink^®^, Gothenburg, Sweden).

Condition	A/Cs/CBD
Material proportions	1 mL of Bioink + 12/24 mg/mL of CBD + 1 mL of distilled water
Printing technique	Extrusion
Print bed temperature	20 °C
Temperature of the nozzle and extruder syringe	20 °C
Print speed	3 mm/s
Printing pressure	10 kPa
First coat drying time	5 min
Dimensions of the printed scaffolding	10 × 10 × 5 mm STL format
Nozzle or extruder size	0.580 mm
Reticulation	CaCl_2_ for 30 min

**Table 3:** FTIR Spectroscopic Comparison of Alginate/Chitosan Control and CBD-loaded Hydrogels.

Wavenumber (cm^−1^)	Functional Group	Control Intensity	CBD-Loaded Intensity	Assignment	Observation
3400–3200	O-H stretching	Strong	Strong	Hydroxyl (Ag/Cs) + Phenolic OH (CBD)	Enhanced and broadened due to additional phenolic groups from CBD
2920	C-H stretching	Medium	Medium	Alkyl chains (enhanced by CBD)	Slight enhancement from CBD alkyl chains
2850	C-H stretching	Medium	Medium	Methyl and methylene groups	Consistent with the CBD aliphatic structure
1631	C=O stretching (Amide I)	Strong	Strong	Carboxyl groups (alginate)	No significant change—matrix integrity preserved
1541	N-H bending (Amide II)	Medium-Strong	Medium-Strong	Amino groups (chitosan)	Unchanged—confirms chitosan structure is maintained
1458	C-H bending	Medium	Medium	Methyl groups (enhanced by CBD)	Enhancement from CBD methyl groups
1385	C-H bending (sym.)	Medium	Medium	Symmetric CH_3_ bending	No significant change
1239	C-O stretching	Strong	Strong	Ether/alcohol C-O bonds	Preserved polysaccharide backbone
1150	C-O stretching	Medium	Medium	Polysaccharide C-O	Matrix structure maintained
1080	C-O-C stretching	Strong	Strong	Glycosidic linkages	Critical polysaccharide bonds preserved
1040	C-O stretching	Medium	Medium	Primary alcohol groups	Hydroxyl functionality maintained
950	C-O stretching	Medium	Medium	Polysaccharide backbone	Structural integrity confirmed
890	Aromatic C-H	Weak	Medium	CBD benzene ring	Key evidence of CBD incorporation

**Table 4:** Comparison of viscosities at different shear rates.

Cutting Speed (s^−1^)	0 mg/mL	12 mg/mL	24 mg/mL	% Increase (12 mg vs. 0 mg)	% Increase (24 mg vs. 0 mg)
0.1	1000.0	2000.0	4000.0	100.0	300.0
1.0	300.0	600.0	1200.0	100.0	300.0
10.0	50.0	100.0	200.0	100.0	300.0
100.0	5.0	10.0	20.0	100.0	300.0

**Table 5:** The power law model describes rheological behavior.

CBD Concentration (mg/mL)	K (Pa·sⁿ)	IC 95% K	n	IC 95% n
0	378	325–419	0.555	0.346–0.600
12	756	654–849	0.555	0.334–0.603
24	1511	1291–1672	0.555	0.343–0.595

## Data Availability

The original contributions presented in this study are included in the article. Further inquiries can be directed to the corresponding authors.

## Abbreviations

The following abbreviations are used in this manuscript:

Ag/Cs	Alginate/Chitosan
CBD	Cannabidiol
